# Early occurrence of inspiratory muscle weakness in Parkinson’s disease

**DOI:** 10.1371/journal.pone.0190400

**Published:** 2018-01-12

**Authors:** Guillaume Baille, Thierry Perez, David Devos, Valérie Deken, Luc Defebvre, Caroline Moreau

**Affiliations:** 1 Department of Neurology and Movement Disorders, Lille University Medical Center, Lille, France; 2 INSERM UMR 1171,University of Lille, Lille, France; 3 Lung Function Department, Lille University Medical Center, Lille, France; 4 Department of Medical Pharmacology, Lille University Medical Center, Lille, France; 5 Department of Biostatistics, Lille University Medical Center, Lille, France; Kobenhavns Universitet, DENMARK

## Abstract

**Introduction:**

In Parkinson’s disease (PD), respiratory insufficiency (including functional and muscle disorders) can impact dysarthria and swallowing. Most studies of this topic have been performed retrospectively in populations of patients with advanced PD. The objective of the present study was to characterize lung function (under off-drug conditions) in early-stage PD patients at baseline and then again two years later.

**Methods:**

Forty-one early-stage PD patients (mean ± SD age: 61.7 ± 7.7; mean ± SD disease duration: 1.9 ± 1.7 years) were prospectively enrolled and compared with 36 age-matched healthy controls. Neurological evaluations and pulmonary function testing were performed in the off-drug condition at the inclusion visit and then two years later.

**Results:**

Pulmonary function testing did not reveal any restrictive or obstructive disorders; at baseline, inspiratory muscle weakness was the only abnormality observed in the PD group (in 53.7% of the patients, vs. 25% in controls; p = 0.0105). The PD patients had a lower mean maximal inspiratory mouth pressure than controls and a lower sniff nasal inspiratory pressure. Two years after the initiation of chronic treatment with antiparkinsonian medications, the maximal inspiratory mouth pressure and the sniff nasal inspiratory pressure tended to be higher. Lastly, overall motor outcomes were not significantly worse in patients with inspiratory muscle weakness than in patients without inspiratory muscle weakness.

**Conclusion:**

Inspiratory muscle weakness seems to be common in patients with early-stage PD, and was seen to be stable over a two-year period. Additional long-term follow-up studies are required to specify the impact of this new feature of PD.

## Introduction

Parkinson’s disease (PD) is the second most frequent neurodegenerative disease [1]. Although patients with PD complain of many non-motor symptoms, the diagnosis of this condition is still based on the observation of motor signs (such as rest tremor, akinesia, and rigidity). When considering non-motor symptoms, pulmonary dysfunction has been described as a dysautonomic sign of PD in several studies; however, the prevalence of this dysfunction has probably been underestimated. The literature data diverge; some researchers have evidenced changes in lung volumes in PD patients (such as an airflow limitation [2,3], a restrictive pattern [4,5,6] or a mixed pattern [7,8]), whereas others have reported ventilatory muscle weakness [9,10]. Furthermore, most studies have been performed in the “on-drug” condition in advanced PD patients (i.e. more than 5 years after disease onset), and we are not aware of any long-term follow-up studies.

According to Braak et al., alpha synuclein deposition and neuron loss start in the caudal part of the brainstem [11]. Given the physiology of respiratory control, structures in the pons and the medulla oblongata might be affected by the initial neurodegeneration in PD [12]. Early alpha synuclein deposition in the nuclei responsible for coordinating ventilation or analyzing the peripheral detection of hypoxemia or hypercapnia might have a harmful impact on respiration. Impaired lung function in PD may be involved in the pathophysiology of axial symptoms such as dysarthria (respiratory muscle weakness, possibly leading to hypophonia [13]) and swallowing disorders (with a shorter period of apnea during the pharyngeal phase of deglutition [14]).

The objective of the present study was to prospectively assess pulmonary function in a cohort of early-stage PD patients at inclusion and then two years later (i.e. after the initiation of chronic treatment with dopaminergic medications). In view of Braak’s model, we hypothesized that pulmonary function would be worse in early-stage PD patients than in healthy controls.

## Methods

In this prospective pilot study, all the participants belonged to the “Prospective Assessment of Dysarthria and Other Dopaminergic and Non Dopaminergic Axial Signs in PD” cohort (PRODYGI-PARK; ClinicalTrials.gov: NCT 02627664) and were included during an inclusion visit between September 2011 and December 2012. All of the patients (i) met the UK Brain Bank criteria [15], (ii) had a Hoehn and Yahr score below 3 [16], and (iii) had been diagnosed and included early in the course of PD (i.e. a disease duration of less than 5 years, as determined by the onset of the first motor symptoms reported by the patient). None of the patients suffered from a concomitant respiratory disease. Likewise, patients with an ear, nose or throat disease (a tumor, an infection or a functional disorder of the vocal cords) or severe cognitive disturbance (as defined by a Mini-Mental State Examination score of less than 24 out of 30 [17]) were excluded. Thirteen of the 41 (32%) patients were had not been treated with parkinsonian medication at inclusion. The remaining 28 (68%) patients had been receiving stable, moderate doses of dopaminergic drugs for at least one month prior to inclusion. With the exception of the neuropsychological assessment, all examinations were performed in the “off-drug” condition. (i.e. at least 12 hours after the last administration of antiparkinsonian medication, typically taken the previous evening).

The study was approved by the local investigational review board (*Comité de Protection des Personnes Nord Ouest IV*, Lille, France: reference: 11/07 2010-A01391-38), and all participants gave their written, informed consent. We compared the patients’ baseline pulmonary function testing (PFT) data with those recorded for a historical cohort of 36 gender- and age-matched (± 5 years) healthy controls and volunteers for a lung examination (mean ± SD age: 61 ± 5.2). All patients were tested in an outpatient clinic.

Clinical examinations were performed by a neurologist with experience in the diagnosis and management of movement disorders. We scored parts I (non-motor aspects of experiences of daily living), II (motor aspects of experiences of daily living), part III (motor examination) and IV (motor complications) of the Unified Parkinson’s Disease Rating Scale (UPDRS [18]). Antiparkinsonian medications were assessed and expressed as the levodopa equivalent daily dose (LEDD) [19].

Pulmonary function testing was performed in Lille University Medical Center’s lung function department. Dyspnea was assessed on the Medical Research Council scale [20], which ranges from 0 (“not troubled by breathlessness except with strenuous exercise”) to 4 (“too breathless to leave the house or breathless when dressing or undressing”). Spirometry and lung volume measurements (nitrogen washout) were measured with a HypAir Compact+® system (Medisoft Group, Sorinnes, Belgium). We measured the total lung capacity (TLC), forced vital capacity (FVC), and the forced expiratory volume in one second (FEV1). Respiratory muscle assessment was also performed with a HypAir Compact+® system, with measurement of the maximal inspiratory mouth pressure (MIP) and the sniff nasal inspiratory pressure (SNIP). The PFT procedures complied with European Respiratory Society’s guidelines on defining an obstructive, restrictive or mixed pattern of respiratory impairment [21]. The respiratory muscle pressures were interpreted with regard to the predicted values published by Uldry et al. [22]. Inspiratory muscle weakness was defined as concomitant changes in the MIP and SNIP (below the lower limit of normal, i.e. the 5^th^ percentile). The PFT was performed at the inclusion visit (V1) for both groups and then two years later (V2) for the PD group only. The neuropsychological examination included the Montreal Cognitive Assessment [23]. Quantitative variables were expressed as the mean ± SD (for normally distributed variables) or the median [interquartile range]. Qualitative variables were expressed as the number (percentage). The normality of the data distribution was assessed using histograms and the Shapiro-Wilk test. A bivariate analysis was performed using Student’s t test for quantitative variables (or a Mann-Whitney U test for non-normally distributed variables) and a chi-squared test for categorical variables (or Fisher’s exact test if the expected cell frequency was <5). An analysis of covariance was used to assess changes over time in clinical parameters for PD patients with or without inspiratory muscle weakness. The change over time in clinical quantitative variables was assessed using Student’s paired t test or (for non-normally distributed variables) a Wilcoxon paired test. Correlations between two quantitative variables were evaluated by calculating Pearson’s coefficient or (for non-normally distributed variables) Spearman’s coefficient. The threshold for statistical significance (two-tailed) was set to p<0.05. All statistical analyses were performed using SAS software (version 9.3, SAS Institute Inc., Cary, NC, USA).

## Results

After a two-year follow-up period, the study population comprised 41 patients (25 men and 16 women; mean ± SD age: 61.7 ± 7.7 years; mean disease duration: 1.9 ± 1.7 years) and was divided into two subgroups (according to the clinical phenotype). There were 15 participants in the “tremor-predominant” group and 26 in the “akinetic-rigid” group (Table 1).

**Table 1:** A comparison between PD patients and healthy subjects.

	PD patients n = 41	Controls n = 36	p
**Age (years)**	61.7 ± 7.7	61 ± 5.2	0.65***
**Gender (M/F)**	25/16	24/12	0.60*
**Phenotype**	- 15 tremor-predominant (36.6%)	NA	NA
	- 26 akinetic-rigid (63.4%)		
**Disease duration (years)**	1.9 ± 1.7	NA	NA
**LEDD (mg)**	304.2 ± 310	NA	NA
**Treatment**	-13 drug-naïve (32%)	NA	NA
	-9 taking levodopa alone (22%)		
	-19 taking levodopa and dopaminergic agonists		
	(46%)		
**MoCA (out of 30)**	27 ± 2.2	NA	NA
**UPDRS part I (out of 16)**	5.1 ± 3.7	NA	NA
**UPDRS part II (out of 52)**	5.9 ± 4.2	NA	NA
**UPDRS part III (out of 108)**	19 ± 8.3	NA	NA
**UPDRS part IV (out of 23)**	0.8 ± 1.1	NA	NA
**Symptomatic patients (MRC≥1)**	17 (41%)	NA	NA
**MRC dyspnea scale (out of 4)**	0.6 ± 0.8	NA	NA
**Tobacco use**	5 (12.2%)	8 (22.2%)	0.24*
**Active smoking**	1	2	NA
**Obstructive pattern**	6 (14.6%)	4 (11.1%)	0.74**
**FEV1/FVC (%)**	75.1 ± 7.2	75.5 ± 5.9	0.80*
**Restrictive pattern**	1	2	NA
**FEV1 (% predicted)**	106.3 ± 13.3	97.7 ± 17.5	0.002***
**FVC (% predicted)**	111.9 ± 14.9	101.6 ± 12.3	0.002***
**TLC (% predicted)**	111.8 ± 17.4	101 ± 14.3	0.004***
**Inspiratory muscle weakness**	22 (53.7%)	9 (25%)	0.011**
**MIP (% predicted)**	75.2 ± 34.2	90.6 ± 26.1	0.035***
**SNIP (% predicted)**	71.8 ± 30.9	89.7 ± 18.6	0.004***

M: male, F: female, LEDD: levodopa equivalent daily dose, MoCA: Montréal Cognitive Assessment, UPDRS: Unified Parkinson’s Disease Rating Scale, MRC: Medical Research Council; FEV1: forced expiratory volume in one second, FVC: forced vital capacity, TLC: total lung capacity, MIP: maximal inspiratory mouth pressure, SNIP: sniff nasal inspiratory pressure. NA: not applicable

* in a chi-squared test

** in Fisher’s exact test

*** in Student’s test

### Comparison of PD patients with controls

The clinical and PFT data are summarized in Table 1. The mean TLC (p = 0.004), the mean FEV1 (p = 0.002) and the mean FVC (p = 0.002) were significantly higher in the PD group than in the control group. Inspiratory muscle weakness was also more prevalent in the patient group than in the control group (p = 0.011). Both the mean MIP and mean SNIP were significantly lower in the patient group than in the control group (p = 0.035 and p = 0.004, respectively).

### Comparison of treated with drug-naïve patients

With the exception of FEV1 (p = 0.03), the drug-naïve and treated PD patients did not differ significantly with regard to the PFT results (Table 2); this was true for the SNIP (p = 0.52), the MIP (p = 0.12), and the proportion of patients with inspiratory muscle weakness (p = 0.51).

**Table 2:** A comparison between drug-naïve and treated PD patients.

	Drug-naïve PD patients n = 13	Treated PD patients n = 28	p
**Age (years)**	61.1 ± 7.6	62 ± 7.8	0.74
**Gender (M/F)**	8/5	17/11	0.96
**MRC dyspnea scale (out of 4)**	0.23 ± 0.44	0.75 ± 0.84	0.06
**FEV1/FVC (%)**	75.7 ± 5.5	74.8 ± 7.9	0.8
**FEV1 (% predicted)**	112.6 ± 13.9	103.4 ± 12.2	0.03*
**FVC (% predicted)**	116.4 ± 12.7	109.7 ± 15.6	0.13
**TLC (% predicted)**	112.6 ± 21.9	111.4 ± 15.3	0.66
**Inspiratory muscle weakness**	6 (46.2%)	16 (57.1%)	0.51
**MIP (% predicted)**	89.3 ± 39	69.3 ± 30.8	0.12
**SNIP (% predicted)**	74.2 ± 31.6	70.8 ± 31.3	0.52

M: male, F: female, MRC: Medical Research Council; FEV1: forced expiratory volume in one second, FVC: forced vital capacity, TLC: total lung capacity, MIP: maximal inspiratory mouth pressure, SNIP: sniff nasal inspiratory pressure.

* in a U Mann Withney test

### The LEDD and the PFT results

For treated PD patients at V1 (n = 28), no correlation was observed between the LEDD on one hand and FEV1 (p = 0.55, r = 0.12), FVC (p = 0.083, r = 0.04), FEV1/FVC (p = 0.82, r = 0.04), TLC (p = 0.33, r = 0.19), MIP (p = 0.11, r = -0.32) or SNIP (p = 0.46, r = -0.15) on the other.

### The motor phenotype and the PFT results

Akinetic-dominant PD patients had a significantly higher FVC (p = 0.05) and a significantly lower FEV1/FVC (p = 0.005), relative to tremor-dominant PD patients (Table 3). There were no other subgroup differences in the PFT results.

**Table 3:** A comparison between tremor and akinetic dominant PD patients.

	Tremor-dominant PD patients n = 15	Akinetic-dominant PD patients n = 26	p
**Age (years)**	60.4 ± 9.2	62.5 ± 6.7	0.4
**Gender (M/F)**	8/7	17/9	0.45
**MRC dyspnea scale (out of 4)**	0.73 ± 0.88	0.5 ± 0.71	0.44
**FEV1/FVC (%)**	79.3 ± 7.6	72.7 ± 5.8	0.005*
**FEV1 (% predicted)**	103.1 ± 13	108.41 ± 13.4	0.26
**FVC (% predicted)**	109.5 ± 13.7	115.3 ± 14.6	0.05*
**TLC (% predicted)**	112.6± 21.9	111.4 ± 15.3	0.7
**Inspiratory muscle weakness**	9 (60%)	13 (50%)	0.54
**MIP (% predicted)**	74.4 ± 41.3	75.6 ± 31.1	0.61
**SNIP (% predicted)**	75.2 ±.28.6	70 ± 32.4	0.46

M: male, F: female, MRC: Medical Research Council; FEV1: forced expiratory volume in one second, FVC: forced vital capacity, TLC: total lung capacity, MIP: maximal inspiratory mouth pressure, SNIP: sniff nasal inspiratory pressure.

* in a U Mann Withney test

### Changes over time at V2

The changes over time in clinical features and PFT data between V1 and V2 are summarized in Table 4. With the exception of the UPDRS part I score (p = 0.096), the MIP (p = 0.055) and the SNIP (p = 0.056), all clinical parameters had significantly worsened after two years of follow-up. The LEDD was significantly higher (p<0.0001), and the FEV1/FVC was significantly lower (p = 0.002). The correlation between the LEDD and changes in FEV1/FVC was not statistically significant (p = 0.55). Patients with a higher LEDD tended to have higher MIP (p = 0.02, r = 0.37) and SNIP (p = 0.05, r = 0.53) values–greater respiratory muscle strength, in other words.

**Table 4:** Changes over time in clinical and PFT parameters between V1 and V2.

	V1	V2	delta	p
**UPDRS I (out of 16)**	5.1±3.7	6.3 ± 3.8	1.2 ± 4.4	0.01*
**UPDRS II (out of 52)**	5.9±4.2	7.2 ± 4.9	1.3 ± 3.7	0.028*
**UPDRS III (out of 108)**	19.0±8.3	23.5 ± 10.0	4.6 ± 8.6	0.002*
**UPDRS IV (out of 23)**	0.8±1.1	2.0 ± 2.0	1.2 ± 2.1	0.001*
**LEDD (mg)**	304.2±310.0	538.5 ± 360.9	234.3 ± 324.2	<0.0001*
**MoCA**	27.0±2.2	27.1 ± 2.2	0.1 ± 1.9	0.80*
**FEV1/FVC (%)**	75.1 ± 7.2	72.2 ± 5.9	-2.7 ± 5.0	0.002*
**FVC (% predicted)**	111.9 ± 14.9	114.7 ± 14.2	1.9 ± 7.5	0.13*
**FEV1 (% predicted)**	106.3 ± 13.3	105.4 ± 15.0	-0.9 ± 6.2	0.36*
**TLC (% predicted)**	111.8 ± 17.4	109.5 ± 16.8	-2.6 ± 17.2	0.36*
**MIP (% predicted)**	75.2 ± 34.2	77.5 ± 22.9	4.0 ± 25.1	0.055**^a^
**Median [IQR]**	68 [53–93]	76 [66–97]	5 [–2–14]	
**SNIP (% predicted)**	71.8 ± 30.9	76.5 ± 17.6	5.2 ± 28.2	0.056^a^**
**Median [IQR]**	71 [50.5–88]	81 [71–90]	8.5 [-2.5–17]	

equivalent daily dose, MoCA: Montréal Cognitive Assessment, FEV1: forced expiratory volume in one second, FVC: forced vital capacity, TLC: total lung capacity, MIP: maximal inspiratory mouth pressure, SNIP: sniff nasal inspiratory pressure.

a: non-significant difference.

* in Student’s paired test

** in a Wilcoxon paired test

### Effect of the inspiratory muscle phenotype on disease outcomes

There was no significant difference between the two subgroups of PD patients (i.e. those with vs. without inspiratory muscle weakness) in terms of the clinical outcome (p = 0.84 for the UPDRS part III score) or the change in LEDD (p = 0.83) (Table 5).

**Table 5:** Comparison (in an analysis of covariance) of clinical changes between V1 and V2 in PD patients with and without inspiratory muscle weakness.

	PD patients with inspiratory muscle	PD patients with normal inspiratory	p
	weakness	muscle strength	
	n = 22	n = 19	
**Change in the UPDRS part I score**	0.3± 4.8	2.2± 3.8	0.94
**Change in the UPDRS part II score**	1.1 ± 4	1.5± 3.4	0.96
**Change in the UPDRS part III score**	4.4± 8.4	5.2 ± 9	0.84
**Change in the UPDRS part IV score**	1.3± 2.5	1.1 ± 1.7	0.35
**Change in the LEDD**	187.2± 371.5	288.8± 258.3	0.83

UPDRS: Unified Parkinson’s Disease Rating Scale, LEDD: levodopa equivalent daily dose.

### The impact of motor fluctuations on PFT

At V2, the UPDRS IV score was seen to be correlated with the MIP (p = 0.005, r = -0.43) and the SNIP (p = 0.05, r = -0.3) but not with FEV1 (p = 0.55, r = 0.12) or FVC (p = 0.68, r = 0.06).

## Discussion

The present study’s main findings were as follows: (i) significant inspiratory muscle weakness (as measured by the maximal inspiratory mouth pressure and sniff nasal inspiratory pressure) can be observed in the early stages of PD, (ii) antiparkinsonian medication does not seem to affect the PFT results, and (iii) the maximal inspiratory mouth pressure and sniff nasal inspiratory pressure tended to be higher at V2 (i.e. after the initiation of chronic treatment with antiparkinsonian medication).

To the best of our knowledge, the present study is the first to have prospectively assessed inspiratory muscle weakness in early-stage PD patients. Interestingly, antiparkinsonian medications may be responsible (at least in part) for the maintenance of the MIP and SNIP values after two years. Although dopamine is not known to increase muscle strength, it might sustain the PFT results by improving muscle coordination.

Inspiratory muscle strength was impaired in PD patients (relative to healthy controls), as characterized by lower % predicted MIP and SNIP values (according to Uldry et al., 1995). Other studies have yielded similar results for the MIP, albeit in later stages of the disease (e.g. a mean disease duration of over 5 years and a Hoehn and Yahr score sometimes higher than 2 [24,25,9,10]). At V1, the statistically significant patient vs. control differences in lung volumes were not clinically significant; this contrasts with the many literature reports of an airway obstruction and, in some cases, a restrictive syndrome [2,3,7,8,26,27,28]. However, these studies did not include patients with promptly diagnosed, early-stage PD; in contrast, the mean disease duration in our study was 1.9 ± 1.7 years. Furthermore, the drug-naïve patients and the treated patients (at V1) did not differ with regard to the PFT data in general and inspiratory muscle weakness in particular, and the LEDD and PFT results were not correlated. Hence, one can suppose that ventilatory dysfunction is part of the pathophysiological process, rather than an effect of treatment with dopaminergic medications. However, these results need to be confirmed in a larger population.

In agreement with other reports [29,30], our patients’ mean UPDRS III scores worsened by 2.1% over two years. The LEDD also increased, and FEV1/FVC decreased significantly. We cannot rule out a role of age in these changes, although there is a lack of detailed data on this topic in the literature. When considering inspiratory muscle strength, the changes over time in MIP and SNIP were non-significant. Factors other than age may explain these results. We did not observe any relationships between changes in pulmonary function and a clinical decline. Increases in the LEDD were not associated with changes in FEV1/FVC. Our present results cannot be directly compared with those of other studies in which lung volume dopasensitivity was assessed following an acute administration of levodopa (with conflicting results) [2,5,6]. However, we observed that changes in the MIP and the SNIP were correlated with an increase in the LEDD. De Bruin et al. observed that the MIP increased after an acute apomorphine injection [24]. Acute levodopa was found to produce an improvement in inspiratory muscle function in anesthetized dogs [31], and dopamine improved diaphragm function during acute respiratory failure in patients with chronic obstructive pulmonary disease [32]. In our cohort, motor fluctuations (mainly induced by levodopa treatment) had a negative impact on MIP and SNIP. Therefore, our results raise the question of whether dopaminergic drugs have a differential effect on ventilatory function, with a potentially positive effect of chronic levodopa administration and a potentially negative impact of acute levodopa intake [33].

The impact of impaired pulmonary function on the cognitive course of PD has never been assessed. In a cohort of 740 PD patients followed up for 6.5 years, the presence of lung function disorders was not a prognostic factor for the occurrence of cognitive disorders [34]. However, the researchers did not objectively measure MIP, SNIP and lung volumes in PFT. We did not observe any changes in cognitive function in our study population—probably because the follow-up period of two years was too short and our cognitive assessment was not detailed enough.

Our study had some limitations. Firstly, PFT was not performed for the control group at V2. However, the results were unlikely to have declined by more than by the predicted values in healthy subjects with normal baseline values. Only Enright et al. highlighted a slight decrease in MIP (of between 0.8 and 2.7 cm/H20 per year) in a cohort of 4443 subjects over the age of 65 [35]. Secondly, the level of effort required by the PFT procedure (which requires voluntary maneuvers) may have had an effect on the quality of the data. Again, the value in the control group was clinically normal. A non-volitional technique (such as transcranial magnetic stimulation) could be used to assess diaphragm strength and respiratory muscle recruitment more specifically in PD. Thirdly, we did not assess the dopasensitivity of the PFT results. Fourthly, very few PD patients had undergone a polysomnographic assessment, although the relationship between PD and sleep disorders is well known. Indeed, respiratory sleep disorders can be the first clinical expression of diaphragm dysfunction. Moreover, obstructive sleep apnea can lead to intermittent hypoxemia and cognitive disorders. Lastly, our follow-up period (two years) may have been too short to evidence an effect of inspiratory muscle weakness on the course of PD.

## Conclusion

Inspiratory muscle strength appears to be impaired in very early-stage PD patients. After two years, it was not clear whether muscle weakness had progressed. Levodopa may have a positive effect on inspiratory muscle strength. Pathophysiological studies are needed to assess the impact of potential respiratory involvement on PD in more detail. The chronic hypoxemia caused by pulmonary dysfunction may notably have a role in the neurodegenerative process, as has already been suggested for amyotrophic lateral sclerosis [36] and Alzheimer’s disease [37]. However, extended follow-up of our cohort will be required to assess the clinical prognosis of patients with early-onset inspiratory muscle weakness.

## Supporting information

S1 File Supporting information: Raw values of the patients (demographical and pulmonary functional testing). (XLS)
